# *Equus caballus* papillomavirus type 2 (EcPV2)‐associated benign penile lesions and squamous cell carcinomas

**DOI:** 10.1002/vms3.1342

**Published:** 2024-01-16

**Authors:** Laura Tuomisto, Jenni Virtanen, Kristel Kegler, Lev Levanov, Antti Sukura, Tarja Sironen, Maria Kareskoski

**Affiliations:** ^1^ Faculty of Veterinary Medicine Department of Veterinary Biosciences University of Helsinki Helsinki Finland; ^2^ Faculty of Medicine Department of Virology University of Helsinki Helsinki Finland; ^3^ Faculty of Veterinary Medicine Department of Production Animal Medicine University of Helsinki Helsinki Finland

**Keywords:** equine papillomavirus 2, histopathology, horse, in situ hybridisation, polymerase chain reaction, SCC

## Abstract

**Background:**

Squamous cell carcinoma is the most common genital, ocular and gastric tumour in horses. *Equus caballus* papillomavirus type 2 (EcPV2) DNA has been detected in several studies in equine penile squamous cell carcinomas (SCCs) and precursor lesions providing evidence of a causal role of EcPV2 in equine genital SCCs. Recently, EcPV2 E6/E7 nucleic acids were also detected in equine gastric SCCs, but further studies are required to determine the role of EcPV2 infection in the pathogenesis of gastric SCC. EcPV2 nucleic acids have been rarely described in ocular SCCs and precursor lesions.

**Objectives:**

To investigate the presence of EcPV2 nucleic acids with polymerase chain reaction (PCR) and in situ hybridisation (ISH) in penile hyperplasias, papillomas and SCCs in horses and to determine whether EcPV2 nucleic acids can be detected in SCCs affecting other locations, including the stomach, ocular tissues and larynx.

**Methods:**

Twenty‐one archival formalin‐fixed paraffin embedded (FFPE) tissue samples, including 12 genital lesions comprising penile hyperplasias, papillomas and SCCs, 6 ocular SCCs, 2 gastric SCCs and 1 laryngeal SCC, were screened by PCR and ISH for EcPV2 E6/E7 DNA and mRNA. Archival FFPE tissue samples (eyelid and penile mucosa and preputium) from six horses without a diagnosis or history of neoplastic or papillomavirus‐associated disease were included as controls.

**Results:**

EcPV2 nucleic acids were detected by PCR and ISH in all genital lesions (12/12) and gastric SCCs (2/2), in two ocular SCCs (2/6) and in one laryngeal SCC (1/1). In control horses, one eyelid sample was positive in PCR but not in ISH. The remaining control samples were negative for EcPV2 E6/E7 nucleic acids in PCR and ISH.

**Conclusions:**

These results further support the role of EcPV2 infection in the development of equine genital SCCs and suggest that EcPV2 infection may also act as a predisposing factor for other SCCs in horses, including gastric, ocular and laryngeal SCCs.

## Graphical abstract

The aims of this study was to investigate presence of equine papillomavirus type 2 (EcPV2) nucleic acids with PCR and ISH methods in benign penile lesions, penile squamous cell carcinomas (SCCs) as well as in SCCs of other locations including stomach, ocular tissues and larynx in horses. We demonstrated presence of EcPV2 nucleic acids in all penile lesions including penile hyperplasias (2/2), papillomas (2/2) and SCCs (8/8) and additionally in gastric (2/2), ocular (2/6) and laryngeal (1/1) SCCs.

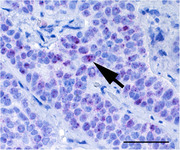

## 1 INTRODUCTION

Papillomaviruses (PVs) are small, circular, non‐enveloped double‐stranded DNA viruses with a circular genome of approximately 8000 bp. PVs belong to the *Papillomaviridae* family, which infect vertebrates worldwide (Greenwood et al., 2020; Lange et al., 2013; Linder et al., 2018; Torres & Koch, 2013). Most PVs are epitheliotropic (Bogaert et al., 2012) and species‐specific (Torres & Koch, 2013). Currently, nine different types of equine papillomaviruses (EcPV1‐9) have been identified with four different clinical manifestations, comprising cutaneous papillomatosis (EcPV1), genital lesions including penile hyperplasia, papilloma and SCC (EcPV2), aural plaques (EcPV3‐6) (Linder et al., 2018; Torres & Koch, 2013) and generalised papillomatosis (EcPV8) (Linder et al., 2018; Peters‐Kennedy et al., 2019). EcPV7 and EcPV9 have been detected in penile masses and in the semen of a horse with a penile lesion, respectively (Lange et al., 2013; Li et al., 2019; Linder et al., 2018; Torres & Koch, 2013). Similar to humans, previous studies suggest that PVs may induce neoplastic processes in horses. In humans, over 200 different human papillomaviruses (HPVs) have been identified. Over 15 HPVs are considered oncogenic high‐risk PVs (hr‐HPVs) and are aetiological agents for different cancer types in several locations, including the cervix, vagina, vulva, penis, anus and the head‐neck region, especially the oropharynx (Alloway et al., 2020; Gheit, 2019; Ramsauer et al., 2019). HPV types 16 and 18 are the most significant types associated with cervical carcinoma in humans (Münger et al., 1989). In horses, EcPV2 nucleic acids are consistently detected with polymerase chain reaction (PCR) and in situ hybridisation (ISH) in penile squamous cell carcinomas (PSCCs) and in related precursor lesions including epithelial hyperplasias, papillomas and carcinomas in situ (Knight et al., 2011; Ramsauer et al., 2019; Scase et al., 2010; Sykora & Brandt, 2017; Torres & Koch, 2013). In contrast to humans, PV‐induced internal genital SCCs have not been described in horses. Additionally, EcPV2 E6/E7 nucleic acids were detected in equine gastric SCCs, of which 7/11 (64%) and 5/11 (45%) were positive with PCR and ISH, respectively (Alloway et al., 2020). There have been other recent case reports of horses with EcPV2‐associated gastric SCC (Porcellato et al., 2020; Straticò et al., 2022).

SCC is a malignant epithelial neoplasm arising from cutaneous and mucosal keratinocytes and is the most common malignant tumour of the skin in horses, accounting for 7%–37% of all cutaneous tumours (Scase et al., 2010; Sykora & Brandt, 2017). SCC can affect the skin, mucosa and mucocutaneous junctions in different locations and is the most common tumour of the external male genitalia, stomach and ocular tissues in horses (Alloway et al., 2020; Bogaert et al., 2012; Newkirk et al., 2014). In male horses, SCCs account for 82.5% of penile and preputial tumours, particularly affecting the glans penis (Van Den Top et al., 2008). SCCs are less commonly reported in female external genitalia (Scase et al., 2010). Gastric SCCs in horses arise typically from the squamous portion of the stomach and usually have a poor prognosis, as the disease is often in an advanced stage at the time of diagnosis (Alloway et al., 2020). Ocular SCCs in horses typically affect the upper and lower eyelids, third eyelid, conjunctiva and limbus (Hendrix, 2005). Early lesions (often referred to as precursor SCC lesions) may manifest as plaques or papular to wart‐like lesions and are histologically compatible with benign hyperplasia or papilloma, which can progress to SCC (Lange et al., 2013; Ramsauer et al., 2019).

The aim of this study was to investigate the presence of EcPV2 nucleic acids in penile hyperplasias, papillomas and genital SCCs in horses and to determine whether EcPV2 nucleic acids can be detected in SCCs affecting other anatomical locations.

## 2 MATERIALS AND METHODS

We selected tissue samples of horses from archived biopsies and necropsies at the Department of Veterinary Biosciences, Faculty of Veterinary Medicine, University of Helsinki, Finland. We included a total of 21 equine cases (3 necropsy cases, 18 biopsies). These consisted of two penile hyperplasias, two penile papillomas, eight penile SCCs, six ocular SCCs, two gastric SCCs and one laryngeal SCC. The cases were submitted between 2007 and 2021. The mean age of the horses at diagnosis was 19 years 6 months, ranging from 7 to 32 years; the age was not reported in four horses. There were 16 geldings, one stallion and four mares. Most of the horses were warmbloods (*n* = 8); other breeds included Icelandic horses (*n* = 5), Shetland ponies (*n* = 2) and Welsh ponies (*n* = 2). No specific breed was recorded for four ponies. Signalments of the horses and diagnostic results are presented in Table 1. Eyelid samples and penile mucosal and preputial tissue samples from six horses submitted for necropsy due to causes other than neoplastic disease were included as controls. Eyelid samples consisted of the lower eyelid including conjunctiva and penile samples were taken at the junction of the inner preputial lamina and the free portion of the penile body. Control horses had no history or diagnosis of any neoplastic disease or PV‐associated disease and their mean age was 12 years 8 months, ranging from 8 to 17 years. Five of the control horses were geldings and one was a stallion. The controls included three warmbloods, two Finnhorses and one Estonian horse. Signalments of control horses and diagnostic results are presented in Table 2.

**TABLE 1:** Signalment, lesion location, histologic diagnosis and PCR and ISH results.

Case No.	Sample collection year	Breed	Age (Y)	Sex	Location of the lesion	Histologic diagnosis	PCR (1st round)	nested PCR (2nd round)	Sequencing performed	ISH
1	2019	Warmblood	15	Gelding	Penis/ preputium	Epithelial hyperplasia	Neg	Pos	Yes	Pos
2	2016	Icelandic horse	23	Gelding	Inner lamina of preputial fold	Invasive SCC	Neg	Pos	Yes	Pos
3	2017	Pony	29	Gelding	Penis/ preputium	Invasive SCC	Neg	Pos	Yes	Pos
4	2014	Icelandic horse	20	Gelding	Glans penis	Invasive SCC	Neg	Pos	Yes	Pos
5	2014	Welsh pony	NR	Gelding	Glans penis	Invasive SCC	Neg	Pos	No	Pos
6	2007	Warmblood	NR	Gelding	Glans penis	Invasive SCC	Neg	Pos	No	Pos
7	2013	Shetland pony	NR	Stallion	Penis	Invasive SCC	Neg	Pos	No	Pos
8	2009	Icelandic horse	19	Gelding	Glans penis	Squamous papilloma	Neg	Pos	No	Pos
9	2018	Pony	10	Gelding	Glans penis	Squamous papilloma/early invasive SCC	Neg	Pos	No	Pos
10	2017	Welsh pony	NR	Gelding	Glans penis	Invasive SCC	Neg	Pos	No	Pos
11	2012	Icelandic horse	26	Gelding	Penis	Invasive SCC	Pos	Pos	Yes	Pos
12	2019	Warmblood	24	Gelding	Penis	Epithelial hyperplasia	Pos	Pos	Yes	Pos
13	2009	Pony	15	Mare	Third eyelid	Invasive SCC	Neg	Neg	No	Neg
14	2020	Warmblood	24	Gelding	Third eyelid	Invasive SCC	Neg	Pos	No	Pos
15	2020	Warmblood	15	Mare	Third eyelid	Non‐invasive SCC	Neg	Pos	No	Neg
16	2021	Warmblood	7	Gelding	Third eyelid	Invasive SCC	Neg	Neg	No	Neg
17	2020	Warmblood	15	Gelding	Third eyelid	Non‐invasive SCC	Neg	Neg	No	Neg
18	2016	Shetland pony	24	Mare	Cornea	Invasive SCC	Neg	Pos	No	Pos
19	2013	Warmblood	19	Mare	Stomach; squamous and glandular mucosa	Invasive SCC	Neg	Pos	No	Pos
20	2008	Pony	17	Gelding	Stomach; margo plicatus	Invasive SCC	Neg	Pos	No	Pos
21	2015	Icelandic horse	32	Gelding	Larynx	Invasive SCC	Neg	Pos	Yes	Pos

NR: not reported; PCR: polymerase chain reaction; ISH: in situ hybridisation.

**TABLE 2:** Control horses with signalment, location of the tissue sample and PCR and ISH results.

Case no.	Sample collection year	Breed	Age (Y)	Sex	Sample location	PCR (1st round)	nested PCR (2nd round)	ISH
22	2022	Warmblood	17	Gelding	Penis‐preputium	Neg	Neg	Neg
					Eyelid	Neg	Neg	–
23	2022	Estonian horse	10	Gelding	Penis‐preputium	Neg	Neg	–
					Eyelid	Neg	Neg	–
24	2022	Warmblood	16	Gelding	Penis‐preputium	Neg	Neg	–
					Eyelid	Neg	Neg	–
25	2022	Finnhorse	12	Stallion	Preputium‐penis	Neg	Neg	Neg
					Eyelid	Neg	Neg	Neg
26	2022	Warmblood	13	Gelding	Penis‐preputium	Neg	Neg	Neg
					Eyelid	Neg	Pos	Neg
27	2022	Finnhorse	8	Gelding	Penis‐preputium	Neg	Neg	Neg
					Eyelid	Neg	Neg	–

Pos: positive; Neg: negative; –: not performed; PCR: polymerase chain reaction; ISH: in situ hybridisation.

### 2.1 Histopathology

Haematoxylin and eosin (H&E)‐stained slides were retrieved from the archives and re‐evaluated to confirm diagnosis. Biopsies included the neoplasm and surrounding tissues. All tissues were fixed in 10% neutral‐buffered formalin for 24 to 72 h and processed routinely to produce 4‐μm‐thick H&E‐stained sections.

### 2.2 DNA extraction and PCR

DNA was extracted from paraffin blocks with RecoverAll Multi‐Sample RNA/DNA Isolation Workflow (Thermo Fisher Scientific, Waltham, MA, USA) according to the manufacturer's instructions. DNA concentration was measured using a Qubit fluorometer (Thermo Fischer Scientific, Waltham, MA, USA). The presence of EqPV2 DNA was assessed with nested PCR. The first round of PCR was performed according to Alloway et al. (2020). PCR products were run on a 2% agarose gel. To confirm the PCR results, the samples with correct‐sized products in the first round (two samples) were purified with a GeneJet PCR purification kit (Thermo Fisher Scientific, Waltham, MA, USA) and sequenced with Sanger sequencing with both primers. Due to the low concentration of DNA acquired from paraffin blocks and the small number of positive samples, a nested round was designed based on Sanger sequences from the first round and published EcPV2 sequences in GenBank. The nested round was carried out with the primers EcPV2_2F (ACCTGAACCTGGTTATGAGAG) and EcPV2_2R (TTTTCCACAGTGGGTGCAG) under the same conditions as the first round. Seven of the products with correct size were purified and sequenced as described. Water was used as a no‐template control. DNA extracted from FFPE tissues of horses without a diagnosis of neoplastic or PV‐associated diseases (case nos. 22–27) were used as negative controls. A synthetic plasmid containing nucleotide sequence 278–543 of EcPV2 (EU503122.1) E6 gene was used as a positive control (GeneArt, Thermo Fisher Scientific, Waltham, MA, USA). Sequences were aligned using MEGA 11, using ClustalW for alignments (Tamura et al., 2021). All sequences were deposited in GenBank under accession numbers OR509031‐OR509037.

### 2.3 In situ hybridisation

In situ hybridisation (ISH) was performed on 4‐μm‐thick FFPE sections using RNAscope® technology (Advanced Cell Diagnostics, Newark, CA, USA) with a probe targeting EcPV2 E6/E7 (cat. 427111) and RNAscope 2.5 HD Reagent Kit‐Red (cat. 322350). The manufacturer's protocol was followed without modifications. FFPE tissues that scored positive by EcPV2 PCR were used as positive controls for ISH. FFPE tissues including eyelid and/or penile/preputial mucosal tissues from selected control horses (case nos. 22, 25–27) scoring EcPV2 PCR‐negative were used as negative controls for ISH (Table 2). The distribution of positive EcPV2 E6/E7 signal in samples was assessed based on cellular morphology using light microscopy.

## 3 RESULTS

### 3.1 Histopathological findings

Two horses were diagnosed with penile hyperplasia; this was characterised by diffuse and marked thickening of stratified squamous epithelium with multifocal rete ridge formation. Occasional keratinocytes, primarily in the superficial stratum spinosum were enlarged with pale eosinophilic to clear cytoplasm and hyperchromatic, sometimes shrunken (pyknotic) nucleus, were interpreted as koilocytes. In the superficial submucosa there was multifocal coalescing, mild to moderate lymphoplasmacytic infiltration admixed with small numbers of neutrophils, eosinophils and macrophages. Both two penile papillomas were characterised by hyperplastic stratified squamous epithelium, which formed papillary projections with a core composed of variable amounts of fibrous connective tissue. Hyperplastic epithelium was multifocally covered by mild to moderate orthokeratotic and parakeratotic hyperkeratosis and occasional swollen keratinocytes (hydropic degeneration) were observed. Within the upper epithelial layers there were multifocal koilocytes. The submucosa showed variable lymphoplasmacytic infiltration. In case no. 9, focal cellular atypia and early invasion was noted, leading to a diagnosis of penile papilloma with possible early invasive SCC.

Penile, ocular, gastric and laryngeal invasive SCCs exhibited relatively similar histological findings. Tumour masses were infiltrative, poorly demarcated, unencapsulated, densely cellular and composed of neoplastic epithelial cells arranged in trabeculae, islands and cords surrounded by moderate amounts of fibrous stroma (Figures 2, 3 and 4A). The cells were polygonal, large and had variably distinct cell borders and moderate to large amounts of eosinophilic cytoplasm. The size of the cells and the amount of cytoplasm increased gradually towards the centre of the neoplastic islands. The nuclei were round to oval and had coarsely stippled chromatin with one to three prominent, amphophilic nucleoli. Multifocally, the neoplastic cells exhibited gradual keratinisation with concentric keratin lamellation (keratin pearls) (Figure 4A), but the degree of keratinisation varied between samples. All SCCs of the third eyelid showed minimal keratinisation, gastric SCCs showed prominent keratinisation and penile, laryngeal and corneal SCCs showed variable keratinisation depending on the area in the same lesion. Anisokaryosis and anisocytosis were moderate to marked; mitoses varied from 9 to 41 per 10 HPF (2.37 mm^2^), and there were no significant differences in these features between different anatomical locations. Neutrophilic or lymphoplasmacytic infiltration (or both) was common within the tumour and in the surrounding tissues. Desmoplasia characterised by extensive fibrosis was frequently observed surrounding the neoplastic cells. Lymphovascular invasion was noted in two cases with invasive ocular SCC (case nos. 13 and 14). Five of the ocular SCCs originated from the conjunctiva of the third eyelid and one originated from the cornea. Two SCCs of the third eyelid did not invade the surrounding tissues and were diagnosed as non‐invasive SCCs (squamous carcinoma in situ) (case nos. 15 and 17). In five ocular SCCs (case nos. 13–16, 18), both penile papillomas (case nos. 8 and 9) and four penile SCCs (case nos. 2, 5, 6, 10) the adjacent epithelium was mild to moderately hyperplastic. In the rest of the samples the perilesional epithelium was normal or not present on the same slides.

### 3.2 PCR results

Amplicons of the expected size were detected in both penile hyperplasias (2/2), in both penile papillomas (2/2), in all penile SCCs (8/8) and gastric SCCs (2/2), in three ocular SCCs (3/6), including one corneal and two third eyelid SCCs and in the laryngeal SCC (1/1). Two of these amplicons (case nos. 11 and 12) were already detected in the first PCR round; the remainder were detected in the nested round. One of the EcPV2 PCR‐positive eyelid samples originated from a control horse (case no. 26). Seven amplicons (two from the first round and five from the second round) were sequenced to confirm their identity and all were shown to be 100% identical to existing EcPV2 E6 sequences in GenBank. Sequences were 98.51%–100.00% similar to each other, with lesions from Icelandic horses harbouring identical sequences (Table 3). Due to the short length (227 bp) of the sequences, a more thorough sequence analysis was not conducted.

**TABLE 3:** Nucleotide similarities (%) between partial E6 sequences from seven of the EcPV2 variants.

	Case no. 4	Case no. 2	Case no. 1	Case no. 21	Case no. 3	Case no. 11
Case no. 2	100.00					
Case no. 1	99.25	99.26				
Case no. 21	100.00	100.00	99.26			
Case no. 3	99.25	99.26	100.00	99.26		
Case no. 11	100.00	100.00	99.26	100.00	99.26	
Case no. 12	98.51	98.53	99.26	98.53	99.26	99.12

### 3.3 ISH results

**FIGURE 1:**
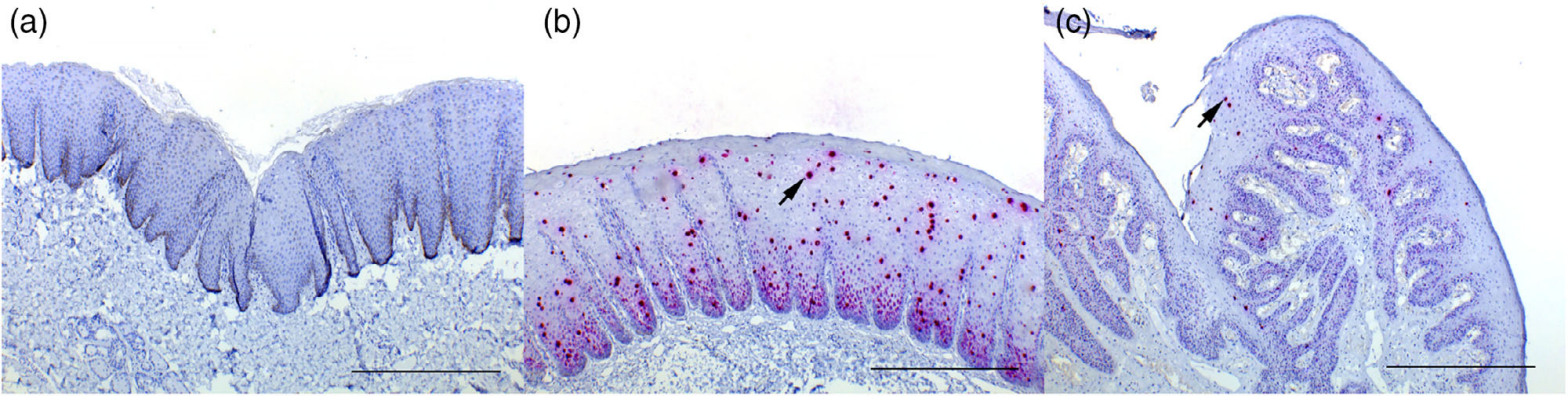
(A) Normal penile mucosa of a control horse no. 25. No hybridisation for EcPV2 E6/E7 probe is present in the epithelium. Bar = 500 μm. ISH, EcPV2 E6/E7 probe. (B) Penile epithelial hyperplasia, horse no. 12. Epithelium is diffusely markedly hyperplastic with formation of rete pegs. Positive hybridisation is present throughout the basal and suprabasal layers. Scattered randomly throughout the epithelium are multifocal intense diffuse nuclear signals (arrow). Bar = 500 μm. ISH, EcPV2 E6/E7 probe. (C) Penile papilloma, glans penis, horse no. 8. Hyperplastic stratified squamous epithelium forming papillary projections supported by a thin fibrous core. Scant positive nuclear signal is present throughout the basal and suprabasal layers with occasional diffuse red nuclear signalling scattered throughout the epithelium (arrow). Bar = 500 μm. ISH, EcPV2 E6/E7 probe.

Positive hybridisation of the EcPV2 E6/E7 probe was detected in all PCR‐positive samples, except for an SCC of the third eyelid (case no. 15) and one eyelid tissue sample from a control horse (case no. 26). In penile hyperplasias and papillomas, positive hybridisation was detected throughout the basal and suprabasal layers, and less frequently in the upper layers and was characterised by primarily multiple nuclear dot‐like signals and an occasional light granular cytoplasmic signal. In both hyperplasias and papillomas, randomly scattered throughout the epithelium, small to moderate numbers of keratinocytes showed an intense diffuse nuclear signal (Figure 1B and C) with variation in signal frequency between different areas in the same lesion.

**FIGURE 2:**
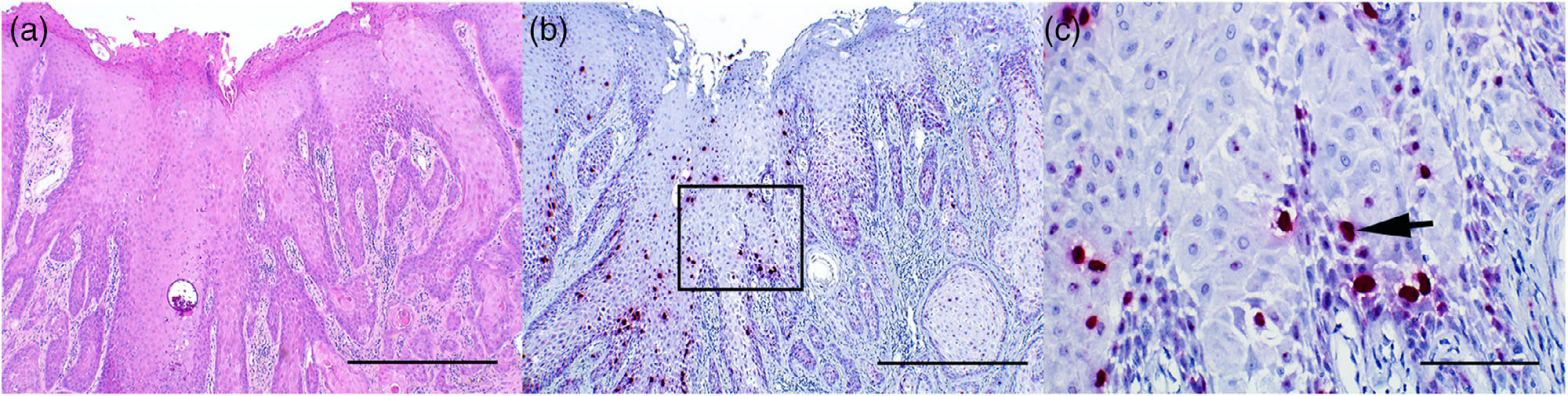
Squamous cell carcinoma, glans penis, horse no 10. (A) Infiltrative, densely cellular mass composed of neoplastic squamous epithelial cells forming trabeculae and islands. Bar = 500 μm. Haematoxylin and eosin (HE). (B) Positive hybridisation is present predominantly in the basal and suprabasal layers with multifocal intense diffuse nuclear signals, which are also apparent multifocally in the upper layers. Bar = 500 μm. ISH, EcPV2 E6/E7 probe. (C) Higher magnification from an area marked with a rectangle in Figure 2B. Intense diffuse nuclear signal (arrow) is multifocally present in the basal and suprabasal layers and also in the upper layers. Bar = 100 μm.

**FIGURE 3:**
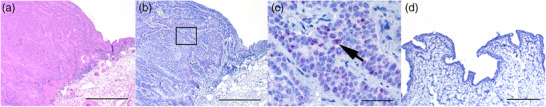
Squamous cell carcinoma, third eyelid (A–C), horse no. 14. (A) Exophytic, infiltrative, densely cellular mass composed of neoplastic squamous epithelial cells arranged in trabeculae and nests. Bar = 500 μm. Haematoxylin and eosin (HE). (B) Positive hybridisation is present predominantly in the basal and suprabasal layers. Bar = 500 μm. ISH, EcPV2 E6/E7 probe. (C) Higher magnification from an area marked with a rectangle in Figure 3B. Multiple nuclear dot‐like signals (arrow) with light granular cytoplasmic signals are present predominantly in the basal and suprabasal layers. Bar = 50 μm. (D) Normal conjunctiva of a control horse no. 22. No hybridisation for EcPV2 E6/E7 probe is present in the epithelium. Bar = 200 μm. ISH, EcPV2 E6/E7 probe.

In penile, ocular and laryngeal SCCs a similar hybridisation pattern was apparent in the basal and suprabasal layers and less frequently in the upper layers and was characterised by multiple nuclear dot‐like signals and an occasional light granular cytoplasmic signal (Figure 3B and C). Small to moderate numbers of keratinocytes throughout the epithelium showed a strong diffuse nuclear signal (Figure 2B and C), but often there was variation in signal frequency between different areas in the same lesion. In three penile SCCs (case nos. 2, 4, 7) and in both ocular SCCs (case nos. 14 and 18) a diffuse nuclear signal was rare and in one penile SCC (case no. 11) it was frequent throughout the tumour. In ocular SCCs only a few cells exhibited a diffuse nuclear signal. In one ocular SCC (case no. 14), positive hybridisation was observed in neoplastic cells within the vessels.

**FIGURE 4:**
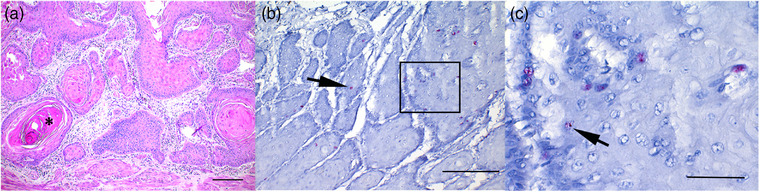
Squamous cell carcinoma, stomach, horse no. 19. (A) Infiltrative, poorly demarcated, densely cellular mass composed of neoplastic squamous epithelial cells forming trabeculae and islands with frequent keratinisation and presence of keratin pearls (asterisk). Bar = 200 μm. Haematoxylin and eosin (HE). (B) Relatively few, randomly scattered epithelial cells primarily in the basal and suprabasal layers of neoplastic islands show positive dot‐like signal (arrow). Bar = 200 μm. ISH, EcPV2 E6/E7 probe. (C) Higher magnification from an area marked with a rectangle in Figure 4B. Low number of cells shows multiple nuclear dot‐like signals (arrow) primarily in the basal and suprabasal layers of neoplastic islands. Bar = 50 μm.

In gastric SCCs the signal frequency in the epithelium was more sparse. In case no. 19, positive hybridisation followed a similar pattern to SCCs of other locations, although it was less frequent and less intense (Figure 4B and C). In case no. 20, positive hybridisation was present multifocally in the basal layers and less frequently in the upper layers and was primarily characterised by a nuclear dot‐like signal and an occasional light granular cytoplasmic signal.

In two ocular SCCs (case nos. 14 and 18), both penile papillomas (case nos. 8 and 9) and three penile SCCs (case nos. 2, 5, 6) the adjacent hyperplastic epithelium showed positive hybridisation. No positive signal was observed in surrounding normal epithelium, if present on the same slides. None of the tissue samples from the control horses showed hybridisation of EcPV2 E6/E7 probe (Figure 1A and D).

## 4 DISCUSSION

The results of this study are consistent with previous reports, supporting the aetiologic role of EcPV2 in equine genital SCCs (Bogaert et al., 2012; Greenwood et al., 2020; Knight et al., 2011; Lange et al., 2013; Scase et al., 2010; Sykora & Brandt, 2017; Torres & Koch, 2013; Zhu et al., 2015). As expected, all penile lesions including penile hyperplasias, papillomas and SCCs were PCR‐positive for EcPV2 DNA. In addition EcPV2 E6/E7 mRNA and DNA were detected with RNA in situ hybridisation in all penile lesions, but not in the normal surrounding epithelium, which supports EcPV2‐induced carcinogenesis.

As described in previous studies (Ramsauer et al., 2019; Zhu et al., 2015), ISH revealed both dot‐like and diffuse nuclear signalling patterns in affected tissues. It has previously been suggested that diffuse nuclear signalling is associated with the presence of both papillomaviral RNA and DNA, which indicates an active EcPV2 life cycle, and this signalling pattern was noted to be more frequent in benign lesions than in malignant lesions (Ramsauer et al., 2019). In our study, multiple nuclear dot‐like signals with a light granular cytoplasmic signal were observed frequently in the basal and suprabasal layers and less so in the upper layers. The frequency of diffuse nuclear signalling varied in both benign and malignant lesions, and there was variation also between different areas in the same lesion, which could be associated with different phases of the EcPV2 life cycle present in the same lesion.

One eyelid tissue sample from a control horse was positive for EcPV2 in PCR but negative in ISH, which may reflect the amounts of viral nucleic acids being very low and not detectable by ISH. No pathological changes were observed in the tissue histologically. Generally, EcPV2 is more commonly detected in SCC samples than in tissues of healthy horses, supporting its role as an oncogenic virus (Jones, 2022; Sykora & Brandt, 2017). One limitation is the low yield of DNA, a common issue with this sample material, which makes it difficult to confirm negative results. Because of the low yield, we used a nested PCR to acquire greater sensitivity, and indeed, most samples were positive only in the nested round. A limitation of nested PCR is a bigger risk of contamination, which should be taken into consideration. Here, appropriate negative controls were used, and PCR and ISH results were consistent with each other, supporting the results (Tables 1 and 2).

In our study, EcPV2 E6/E7 nucleic acids were also detected in gastric, ocular and laryngeal SCCs. Although only two gastric SCCs were included in this study, these findings are consistent with previous reports suggesting an association between EcPV2 and gastric SCCs. This emphasises the need for further research to confirm a causal role of EcPV2 infection in gastric SCC. In contrast to other lesions, both gastric SCCs showed relatively sparse and less intense hybridisation of EcPV2 E6/E7 probe. Our results differ from studies on human gastric SCCs, which are currently not proven to be associated with HPV infection (Alloway et al., 2020).

We detected EcPV2 in two out of six ocular SCCs with both PCR and ISH and in one out of six with only PCR. In our study the ISH method revealed a similar hybridisation pattern as in other SCCs, showing positive nuclear and cytoplasmic dot‐like signals most prominently in the basal and suprabasal layers with rare diffuse nuclear signal. Although some suggested predisposing factors for ocular SCCs include increased ultraviolet radiation and lack of skin pigment, the exact pathogenesis of ocular SCCs is currently unclear (Hendrix, 2005). In 2020, EcPV2 nucleic acids were identified with ISH in 8 out of 68 horses (12%) in eyelid samples collected from asymptomatic Western Canadian horses (Greenwood et al., 2020), but not in ocular/periocular SCCs (Greenwood et al., 2020). Previously EcPV2 DNA has been detected in a periorbital SCC (Kainzbauer et al., 2012), and in two periocular SCCs (Miglinci et al., 2023). However, EcPV2 associated lesions in ocular tissues seem to be rare (Greenwood et al., 2020; Newkirk et al., 2014; Scase et al., 2010; Sykora & Brandt, 2017). Based on scattered reports of EcPV2 associated SCCs and our findings, underlying EcPV2 infection should be considered as a possible predisposing factor for ocular SCCs. Recently, DNA of equine herpesvirus types 2 and 5 (EHV2, EHV5) and asinine herpesvirus type 5 (AsHV5) have been detected in ocular SCCs and precursor lesions, but also relatively frequently in ocular swabs obtained from healthy horses, and the possible role of herpesviruses in the development of SCCs is yet to be determined (Miglinci et al., 2023). Unfortunately, our sample size was small and further studies are required to investigate the association of EcPV2 infection with ocular SCCs.

Additionally, we identified EcPV2 nucleic acids in a laryngeal SCC of one horse. Previously, EcPV2 DNA was detected in a guttural pouch SCC of a horse with associated laryngeal and laryngotracheal SCCs and laryngeal papillomatous lesions and in a laryngopharyngeal SCC of a horse (Hibi et al., 2019; Lale et al., 2023). Additionally EcPV2 DNA has been recently detected in subsets of head‐and‐neck SCCs (HNSCC) in horses (Miglinci et al., 2023; Strohmayer et al., 2022). In humans, oropharyngeal SCCs are most often associated with HPV type 16 (Hibi et al., 2019; Zaravinos, 2014). In our study, no other concomitant EcPV2‐associated lesions were reported in the same horse, although there have been a few reports of horses showing two separate EcPV2‐associated lesions in different locations (Bogaert et al., 2012; Lale et al., 2023; Porcellato et al., 2020). However, in cases of multifocal manifestation of EcPV2‐associated carcinomatous lesions, it often remains unclear whether this is due to metastatic disease or just coincidental development of the lesions.

The oncogenic mechanisms of EcPV2 have not yet been described in detail, but it is believed that both E6 and E7 oncogenes, which are the major viral oncoproteins of HPVs, may also play a role in carcinogenesis in horses. In previous studies, EcPV2 DNA has also been detected in asymptomatic horses, indicating that the presence of viral DNA alone is not pathological and that the carcinogenetic mechanisms are likely more complex (Bogaert et al., 2012; Cappelli et al., 2022; Fischer et al., 2014; Greenwood et al., 2020). This is similar to cervical HPVs, which often lead to asymptomatic infections that may eventually be resolved by the host immune system (Gheit, 2019). In our material, all genital tissue samples from control horses were negative for EcPV2 in both PCR and ISH. SCCs are invasive tumours that compromise the health of affected horses. SCCs typically affect elderly horses, as seen in our study (Lale et al., 2023; Sykora & Brandt, 2017; Van Den Top et al., 2008). In our study population, the mean age of horses with SCC of any location was 20 years with most of the horses being warmbloods (38%), followed by Icelandic horses (24%), Welsh ponies (9.5%) and Shetland ponies (9.5%). A relatively high proportion of Icelandic horses with genital SCCs and precursor lesions has been reported also in earlier studies (Ramsauer et al., 2019; Sykora & Brandt, 2017; Zhu et al., 2015), with specific EcPV2 E6 variant detected in Icelandic horses imported from Iceland, indicating geographical distribution of some EcPV2 E6 gene variants (Lange et al., 2013; Sykora & Brandt, 2017). We detected 100% similarity of sequenced PCR products in Icelandic horses relative to each other, but unfortunately more thorough sequence analysis could not be conducted due to the short length of the PCR product. Therefore we could not establish whether this EcPV2 variant represents the same variant detected in Icelandic horses in previous studies. Tumours require surgical excision, which may be difficult due to their challenging location, advanced disease or both. No prophylactic therapies are currently available; accordingly, investigating potential predisposing factors is essential. Transmission of EcPV2 has not been thoroughly investigated, but infection via direct contact, contaminated fomites, smegma and insects has been suggested (Greenwood et al., 2020; Scase et al., 2010; Sykora & Brandt, 2017; Torres & Koch, 2013). To prevent infection and to develop therapeutic or prophylactic protocols, it is important to gather information on transmission routes, oncogenic mechanisms and impact of EcPV2 infection on prognosis. Currently EcPV2 appears to be the most clinically relevant equine PV, as it is associated with carcinogenesis of genital SCCs and possibly also SCCs of other locations.

## AUTHOR CONTRIBUTIONS

Maria Kareskoski, Kristel Kegler and Antti Sukura contributed to study design and reviewing the manuscript. Maria Kareskoski and Kristel Kegler contributed to data collection. Laura Tuomisto contributed to histological examination of the samples, interpretation of ISH results and manuscript preparation and assisted with DNA extraction. Jenni Virtanen performed DNA extraction and PCR, interpreted PCR results and contributed to manuscript preparation. Lev Levanov prepared the positive control for PCR. All authors have read and approved the final manuscript.

## FUNDING

This study was supported by the Finnish Veterinary Foundation.

## CONFLICT OF INTEREST STATEMENT

The authors declare no conflict of interest.

## ETHICS STATEMENT

Ethical approval was not required for this study due of the use of archived samples submitted to pathological examination in clinical cases. The Finnish legislation (Act on the Protection of Animals Used for Scientific or Educational Purposes 497/2013) does not require the study to be reviewed or approved by an ethics committee as no samples were collected from animals specifically for this study.

## INFORMED CONSENT

Informed consent was not acquired from the animal owners, as the study involved only archival animal samples that were handled anonymously.

### PEER REVIEW

The peer review history for this article is available at https://www.webofscience.com/api/gateway/wos/peer‐review/10.1002/vms3.1342.

## Data Availability

All data generated or analysed during this study are included in this published article and in GenBank under accession numbers OR509031‐OR509037.
